# The Laws of Health in Relation to Mind and Body: A Series of Letters from an Old Practitioner to the Patient

**Published:** 1851-11

**Authors:** 


ARTICLE IV.

*The Laws of Health in relation to Mind and Body : A series of Letters from an old practitioner to the patient.* By LEINEL JOHN BEALE, M. R. C. S. pp. 256 duodecimo. Philadelphia: Blanchard & Lee, 1851. (From the Publishers, through S. C. Griggs & Co.)

This work treats of a subject which is ot the utmost importance to mankind at large and is not designed exclusively for the Professional reader. However, as the Physician should always be posted up on the subject of Hygiene, that he may be able to give good and wholesome instruction to his patients and friends, it will be found worthy of a place in every Medical library. Physicians are too negligent of this subject generally. Few of them conceive it to be their duty to teach people how to prevent disease, being wholly satisfied with efforts to remedy their maladies after they have brought them upon themselves.

It is plain, however, that the old saying, that prevention is better than cure, is founded upon the true philosophy, and therefore the highest mission of the minister of health is to prevent sickness, pain, disease, and death by guarding against those violations of the laws of nature upon which they generally depend.

People are too apt to attribute to the dispensations of Divine Providence the ills that afflict them, especially sickness and unhealthy and shattered constitutions, when a little knowledge of the laws of health will generally show that their own imprudence has been the cause of such difficulties.

There are two reasons why this error prevails to so great an extent. The first is, thatth e people regarding their sickness as Providential and look no further for its source. The second is, that Physicians do not inculcate among them the doctrines of the laws of health and a knowledge of the necessity of obeying them, perhaps because there is no good pretext whereon to attach a fee, and Doctors can as illy afford to work for nothing as any other class of community.

However, it will cost our friends but little effort to advise people to buy the book before us, urging as a most potential argument—one which will in many instances, at least, be convincing, that by studying it, they may save many Doctor's bills.

